# Screening for Postpartum Depression in Well-Baby Care Settings: A Systematic Review

**DOI:** 10.1007/s10995-016-2088-8

**Published:** 2016-08-12

**Authors:** Angarath I. van der Zee-van den Berg, Magda M. Boere-Boonekamp, Maarten J. IJzerman, Riet M. E. Haasnoot-Smallegange, Sijmen A. Reijneveld

**Affiliations:** 1 Department Health Technology and Services Research, Institute of Innovation and Governance Studies, University of Twente, Ravelijn Building Room RA 5260, PO Box 217, 7500 AE Enschede, The Netherlands; 2 Department of Preventive Child Health Care, Municipal Health Services GGD Twente, Enschede, The Netherlands; 3 Department of Health Sciences, University Medical Center Groningen, University of Groningen, Groningen, The Netherlands

**Keywords:** Postpartum depression, Screening, Well baby care, Preventive child health care, Effectiveness, Systematic review

## Abstract

*Introduction* Postpartum depression (PPD) is a mental health problem frequently experienced by mothers in the first year postpartum. Early detection and treatment can help to reduce its negative effect on the development of the newborn child. Well-baby care (WBC) is a promising screening setting for early detection of PPD. This systematic review investigates the evidence of the effectiveness of screening for PPD in WBC settings regarding mother and child outcomes. *Methods* Three electronic databases were searched: SCOPUS, PsychINFO and CINAHL. Two reviewers independently performed the study selection. Data extraction was based on a predefined data extraction form. *Results* Six studies were included; a quality assessment rated two studies as strong and four as weak. Four studies measuring outcomes at process level showed improvement in detection, referral and/or treatment rates. Four studies, including the two strong ones, where screening and enhanced care were combined, showed improvements in the Edinburgh Postnatal Depression Scale scores of the mothers in the intervention groups. No improvements were reported on other outcomes at parent level or at child level. At child level, weight was the only outcome that was measured. *Discussion* This review provides limited yet positive evidence for the value of screening for PPD in a WBC setting. The outcomes are comparable with studies on screening for PPD in general. The evidence that we found is very promising but the small number of available studies shows a need for additional high-quality studies, to strengthen the evidence regarding the potential benefits of screening in a WBC setting.

## Significance

*What is already known on this subject?* Postpartum depression has a high prevalence and its early detection and treatment improves the prognosis of both mother and child. Screening for postpartum depression may be valuable to improve detection and mother and child outcomes, if implemented in the right setting.

*What this study adds?* This review supplies an overview of the current evidence on the value of screening for PPD in a well-baby care setting. The evidence found is limited but promising; it shows that screening in WBC leads to higher detection, referral and treatment and, when combined with enhanced care, to improvement in lowering depression scores.

## Introduction

Children’s early social-emotional development affects their mental health during their entire life-course. The parents’ mental health problems can affect this development negatively. One of the most frequent mental health problems that mothers encounter after delivery is postpartum depression (PPD). An analysis of 28 prevalence studies showed that 7.1 % of women suffer from major depression in the first 3 months postpartum. When minor depression was included, the prevalence increased to 19.2 % (Gavin et al. 2005). Children of mothers who had experienced PPD have more difficulties in their cognitive, social-emotional and language development, and have higher levels of internalizing and externalizing behavior, as well as general psychopathology later in life (Goodman et al. 2011; Kingston and Tough 2012; Brand and Brennan 2009). Early treatment of maternal PPD may reduce these problems (Wan and Green 2009; Sohr-Preston and Scaramella 2006).

Depression can be treated effectively in several ways (O’Hara and McCabe 2013), but many cases of PPD remain undetected, partly because mothers face barriers to discuss their feelings (Liberto 2012) and partly because the professionals they encounter do not recognize the symptoms or fail to discuss them (Heneghan et al. 2000). Therefore, several articles on PPD advocate incorporation of screening in public healthcare (Gavin et al. 2005; Liberto 2012). Well-baby care (WBC) may be a very promising setting for early detection of maternal PPD as this setting provides routine check-ups during the first year after delivery (Gjerdingen et al. 2011). The intention of WBC is to monitor the child’s development and health, including the wellbeing of the parents. Examples of systems supplying this care are: the well-child care in the United States, health visitors in the United Kingdom, Child and Family Health care in Australia and preventive child health care systems in various European countries. Systems providing WBC often have large coverage. In some countries, WBC is being delivered to 95–99 % of newborn children (van den Heuvel et al. 2013), thereby also reaching the majority of postpartum mothers.

A few reviews on the efficacy of screening for PPD are available (Myers et al. 2013; Hewitt et al. 2009), but none of these specifically address the value of screening in a WBC setting. We therefore systematically reviewed the evidence on the effectiveness of screening for PPD in WBC compared to no screening, regarding mother and child outcomes and report our findings here according to the Preferred Reporting Items for Systematic reviews and Meta-Analyses (PRISMA) Statement (Liberati et al. 2009).

## Methods

### Search Method

A search was performed by the first author (A.Z.-B.) in three electronic databases: Scopus (including all the citations in PubMed and Embase from 1996), PsychINFO and CINAHL. We searched the databases for publications up to May 2014. The search strategies were based on the MESH-terms (MEDLINE thesaurus) available for the subject and the key terms extracted from the background literature. Three main concepts were combined and fed into the search engine: postpartum depression, early identification, and well-baby care setting.

As the subject is related to several research areas (psychiatry, child development, primary health care, women‘s health), we added a number of synonyms for each concept. We created several alternative terms for the well-baby care setting as the nature of this kind of setting varies from country to country. Full details of the search strategy in Scopus are reported in Appendix 1. We used the same search strategy for PsychINFO and CINAHL, except for the exclusion of subject areas as these databases do not have this option.

### Selection Process

Two of the authors, A.Z.-B. and M.B.-B., independently assessed the eligibility of the resulting publications in three rounds. The first selection was based on the title. Next, the abstracts of the selected articles were reviewed according to the inclusion and exclusion criteria (Table 1), based on the PICOTS categories (Population, Intervention, Comparators, Outcomes, Timing and Setting). In the final round, the selected articles were judged after full-text-reading. Selected articles that appeared to be reviews were hand searched by one reviewer, A.Z.-B., for additional references. In each stage of the selection process, the reviewers used one of three response options to indicate their opinion as to whether an article should go to the next stage; “yes”, “no”, and “maybe”. The outcomes of the two independent reviewers were compared before proceeding to the next stage. Titles, abstracts and articles with differing opinions were discussed and reread if necessary. An independent third reviewer could be consulted to resolve remaining disagreements, but this proved to be unnecessary. The author of one article (Yawn et al. 2012) was contacted to obtain more information on the setting before deciding on its inclusion.

**Table 1:** Inclusion and exclusion criteria

Study Characteristics	Inclusion Criteria	Exclusion Criteria
Population	Women up to 12 months postpartum	–
Intervention	Isolated screening or screening as a part of a more comprehensive prevention or intervention strategy Screening for postpartum depression using a validated screening instrument for depression	Interventions without a screening component Screening using a non-validated instrument
Comparators	Usual care without a screening instruction protocol or without specific attention for PPD Screening under different conditions (e.g. setting, timing) or with another validated instrument	Studies with no control group to compare the effectiveness of the screening
Outcomes	*At least one of the following outcomes* Validated diagnostic instruments for depression Rates of referral for symptoms of depression, rates of positive diagnosis, and/or implemented treatment Validated measures of maternal well-being, health-related quality of life, parenting Validated measures of child health and development Maternal and/or child health system resource utilization, including number of visits and estimates of total and attributable costs	Reported outcomes provide no information on the effects of the screening
Timing	Screening for depression (at least partly) within the first 12 months postpartum	Screening for depression only during pregnancy
Setting	Offering routine contact with a healthcare professional in the first year postpartum to check the health and development of the child Serving the general population Study located in a high-income economic country as defined by the World Bank	Clinical setting Setting exclusively addressing the woman and not the child Study located in a non- high-income economic country as defined by the World Bank
Study design	Randomized controlled trial Observational study with comparator (prospective or retrospective) Sample size ≥100 subjects Rcts all sample sizes Systematic reviews	Nonsystematic review, Case series, case report, editorial, letter
Report criteria	Article in English, Dutch, German or French Peer-reviewed article Relevant systematic review, meta-analysis	Article in a language other than English, Dutch, German or French No abstract/full text found

**Fig. 1:**
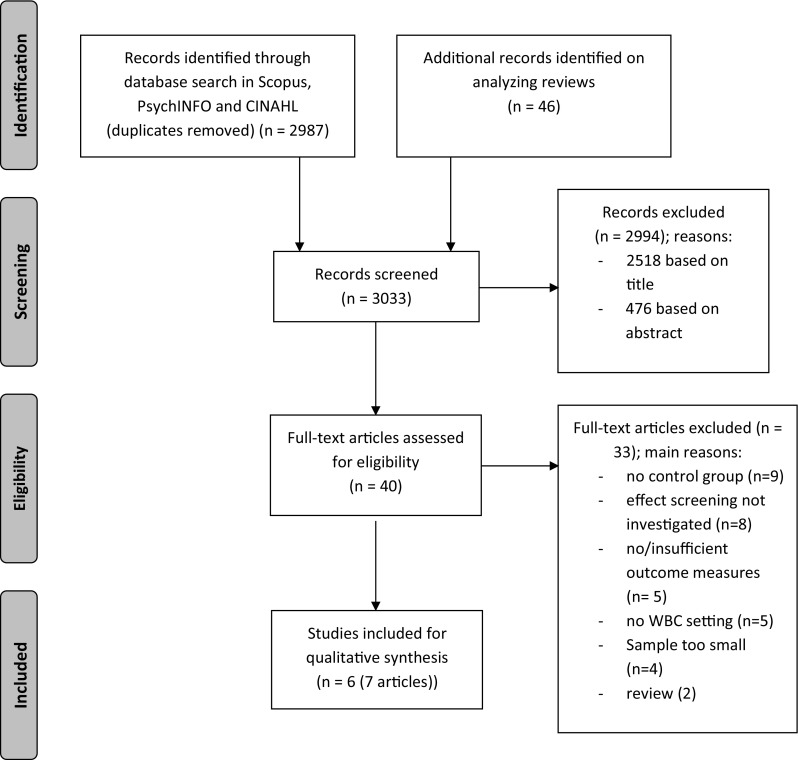
Flow diagram of study selection

A flow diagram of the selection procedure is shown in Fig. 1. Seven articles, concerning six individual studies, met the inclusion criteria and were used in this review.

### Quality Assessment

To assess the quality of the included studies, the reviewers independently applied the Quality Assessment tool for Quantitative Studies, developed by the Effective Public Health Practice Project (EPHPP) (Armijo-Olivo et al. 2012). Studies were rated on six aspects: selection bias, study design, confounders, blinding, data collection method, withdrawals and dropouts. The aspects were explored by answering guiding questions and were next rated according to established criteria, e.g. for an aspect like data collection methods, rating depended on the validity and reliability of the data collection tools. A study received a strong global rating when none of the aspects were weak, a study with one weak aspect was rated as moderate, and two or more weak aspects resulted in a weak global rating. Differences in quality ratings were discussed and agreement was reached by critically applying the criteria again. In addition to the standard EPHPP scoring, possible study specific biases were investigated by comparing method and result sections on contradictions and missing data.

### Data Synthesis

One reviewer (A.Z.-B.) extracted the data from the six selected studies using a predefined data extraction form, including the results of two articles by Glavin et al. (2010) and Glavin (2012); they were compared but there were no conflicting or contradicting data. The data categories are presented in Table 2. The authors of all the included studies were approached for more information on certain aspects, like setting or population; three out of six authors responded and answered our questions. We described the differences and similarities of the studies in terms of setting, population, the intervention applied including specific screening aspects like instrument and timing, and the used outcome measures. After presenting the results of the quality assessment, a narrative synthesis was undertaken. The included studies were reviewed for a shared summary effect measure like risk ratio (RR) or odds ratio (OR), expressing the effect of screening on primary outcomes such as an improvement of depression scores. The extracted data were not pooled or analyzed statistically because of the small number of studies, the differences in the compared interventions, and the heterogeneity of the outcome measures and time horizons.

**Table 2:** Main characteristics of the included studies (N = 6)

References, Country	Study design, blinding	Setting	Sample description, participation and dropouts	Intervention and control conditions	Outcome measures	Main results
Gerrard et al. (1993), England	A pre- and post- design, no blinding	Health visitors in six sectors, some GP-attached and others geographically based	*Sample* Pre-training: mothers 20–26 weeks postpartum in the caseload of the untrained health visitors Post-training: mothers 6–8 or 10–12 weeks postpartum in the caseload of the trained health visitors *N* • Pre-training group: 1008 • Post-training group: 1001	*Intervention* • Screening with EPDS during regular health visits at 6–8 weeks test and/or 10–12 weeks • 4–8 non-directive counseling visits • Health visitors received up to 10 training sessions including education on PPD, use of the EPDS, non-directive counseling skills and prevention of PPD *Control* • Standard service provided by the health visitors; no screening, no training	*Primary (mother level)* • EPDS at 6 months postpartum	*Primary (mother level)* • Improvement of the median score on the EPDS at 6 months postpartum from 7 in the pre-training group to 5 in the post-training group • Decrease in prevalence of EPDS score ≥12 at 6 months postpartum from 19.3 % (pre-training) to 9.8 % (post-training)
Chaudron et al. (2004), New York State, United States	A pre- and post- design, no blinding	Large pediatric primary care practice at the University of Rochester Medical Center	*Sample* Randomly selected child medical records *N* • Before initiation of screening: 110 • After initiation of screening: 110	*Intervention (after):* • Screening with EPDS during each well-child visit in the child’s first year, performed by a pediatric nurse practitioner or pediatrician • Screening considered positive with EPDS ≥10 *Control (before)* • No screening during the well-child visits	*Primary (process level)* • Documentation of depression or depression symptoms • Documentation of referrals to social worker or other providers	*Primary (process level)* • Increase in documentation of depressive symptoms: 1.6–8.5 % • Increase in social worker referrals: 0.2–3.6 %
Glavin (2012), Glavin et al. (2010), Norway	A quasi-experimental post-test study with non-equivalent groups, no blinding	Well-baby clinics of 2 municipalities	*Sample* 89.6 % of 2508 women with a live-born child delivered in 2005–2006 *Inclusion criteria* > 18 years old Able to read and understand Norwegian Not undergoing depression treatment *N* • Intervention group: 1806 • Control group: 441	*Intervention* • Home visit 2 weeks postpartum with increased focus on maternal mental health • One supportive counseling session by the public health nurse after mothers completed the EPDS at 6 weeks postpartum • Supportive counseling sessions for depressed mothers with EPDS ≥ 10 and judged as having PPD by the public health nurse • Openness about mental health issues at every visit • System of referral for further treatment *Control* • Standard service provided by the well-baby clinics	*Primary (mother level)* • EPDS at 6 weeks, 3, 6 and 12 months postpartum *Secondary (mother level)* • PSI at 12 months postpartum	*Primary (mother level)* • OR for depression (EPDS ≥ 10) in intervention group at 6 weeks: OR 0.6 (95 % CI 0.4, 0.8), 3 months: OR 0.4 (95 % CI 0.3, 0.6), 6 months: OR 0.5 (95 % CI 0.3, 0.8) and 12 months: OR 0.6 (95 % CI 0.4, 1.0) • Stronger improvement of EPDS scores at 3, 6 and 12 months of mothers with a ≥10 score on the EPDS 6 weeks after birth, effect size 6 weeks to 12 months: 0.53 *Secondary (mother level)* • Marginally lower level of parenting stress at 12 months: statistical significance only on the Health subscale (PSI)
Leung et al. (2011), Hong Kong	RCT, individual randomization, blinding of participants and nurses	Maternal and Child Health Centers (MCHCs)	*Sample* 83.7 % of 552 mothers of 2 month old children visiting the MCHCs *Exclusion criteria* Non-local residents Not using the Chinese language Participating in other PPD screening programs Receiving psychiatric treatment *N* • Intervention group: 231 • Control group: 231	*Intervention* • EPDS 2 months after birth • Screening considered positive with EPDS ≥ 10 or positive answer on Q10 (suicidal ideation) *Control* • No screening using the EPDS *Intervention and control (same procedures in both groups to mask allocation)* • Clinical assessment by one MCH nurse at 2 months blind to participant’s group status and scores • Referral of screen-positive women or women clinically assessed as depressed, to another MCH nurse (blind to participant’s group status and scores) for further exploration of the condition and non-directive counseling • Recommendation by MCH nurse for further MCH nurse counselling or community psychiatric team referral	*Primary (process level)* • Screen-positives rates • Treatment rates *Primary (mother level)* • EPDS at 6 months postpartum *Secondary (mother)* • EPDS: 18 months GHQ-12, PSI, CKMSS at 6 and 18 months • Number of doctor visits *Secondary (child level)* • Body weight at 6 and 18 months	*Primary (process level)* • Screen-positives: 29 % (67/231) in the intervention group (I) versus 6.0 % (14/231) clinically assessed as probably depressed in the control group (C) • Received treatment: 23.8 % (55/231) in I, 4.8 % (11/231) in C *Primary (mother level)* • EPDS at 6 months ≥10: 13 % of the mothers in I, 22.1 % of the mothers in C (RR 0.59 (95 % CI 0.39–0.89)) • Number Needed to Treat = 25 (after adjustment for positive predictive value of the EPDS) *Secondary (mother/child level)* • More doctor visits in I compared to C (*p* = 0.039) • No difference in all other outcome measures at 6 and 18 months
Yawn et al. (2012), 21 states, United States	Cluster RCT, randomization of practices, no blinding	Family medicine research network practices	*Sample* 97.7 % of 2398 women receiving continuing care at 28 family practices *Inclusion criteria* English or Spanish speaking ≥18 years 5–12 weeks postpartum *N:* • Intervention group: 1353 • Control group: 990	*Intervention* • Set of tools for the practices to facilitate diagnosis, follow-up, and management of PPD • Access to EPDS and PHQ-9 scores (filled in by the mothers 5–12 weeks postpartum) • EPDS ≥ 10 followed by the PHQ-9, evaluated by the physician • Mother considered depressed when PHQ-9 ≥10, confirmed by physician evaluation *Control:* • Usual care • No access to the EPDS and PHQ-9 scores	*Primary (mother level)* • Decrease in PHQ-9 score from baseline to 6 or 12 months postpartum *Secondary (mother level)* • Changes from baseline to 12 months postpartum in PSI and DAS-6 scores • Rates of PPD diagnoses, therapy initiation and referrals registered in the medical record	*Primary (mother level)* • 12 months: OR for a ≥5-point drop in PHQ-9 score between baseline and 12 months: 1.82 (95 % CI 1.14–2.91), adjusted OR: 1.74 (95 % CI 1.05–2.86) *Secondary (mother level):* • No relation between intervention and changes in the PSI or the DAS-6 from baseline to 12 months
Carroll et al. (2013), Indiana, United States	RCT, no blinding	Main primary clinic	*Sample* Mothers of 3520 children aged 0 to 15 months between October 2007 and July 2009 *N* • Intervention group PSF: 1167 • Intervention group JIT: 1167 • Control group: 1186	*Intervention* • Validated 2-question screening tool every 3 months, with one or two positive answers intervention followed: – PSF-group: automatic reminder alerting the physician to the risk and recommending assessment for depression – JIT-group: the same reminder as the PSF-group plus two ‘just in time‘ handouts (JIT): 1. PHQ-9 2. Educational handout with information about maternal depression and community resources for treatment *Control* • No questions adapted in the pre-screening form • Generic reminder on depression presented to the physician	*Primary (process level)* • Registered suspected depression (in the decision support and electronic medical record system) • Answers on the 2-question screening (depressed mood or signs of anhedonia) • Documentation of rate of referral	*Primary (process level)* • Registered depressed mood: PSF-group: 8.8 % (OR 7.93, 95 % CI 4.51 to 13.96), JIT-group: 8.7 % (OR 8.10, 95 % CI 4.61–14.25), control group: 1.2 % • Registered signs of anhedonia: PSF-group: 5.1 % (OR 12.58, 95 % CI 5.03–31.46), JIT-group: 5.2 % (OR 13.03, 95 % CI 5.21–32.54), control group: 0.4 % • Rate of referral: control group: 1.2 %, PSF-group and JIT-group: 2.4 % (OR 2.06, 95 % CI 1.08–3.93)

*RCT* randomized controlled trial, *GP* general practitioner, *MCHC* Maternal and Child Health Center, *EPDS* Edinburgh Postnatal Depression Scale, *PPD* postpartum depression, *MCH* maternal and child health, *PHQ-9* Patient Health Questionnaire, *PSF* pre-screening form, *JIT* ‘just in time‘ handout, *PSI* Parenting Stress Index, *GHQ-12* 12-item General Health Questionnaire, *CKMSS* Chinese Kansas Marital Satisfaction Scale, *DAS-6* Dyad Adult Satisfaction short form, *OR* odds ratio, *CI* confidence interval, *RR* risk ratio

## Results

### Setting and Population

The characteristics of the six included studies are presented in Table 2. The settings of the studies (Yawn et al. 2012; Glavin 2012; Glavin et al. 2010; Chaudron et al. 2004; Leung et al. 2011; Carroll et al. 2013; Gerrard et al. 1993) differ in location and the professionals performing the screening. In the studies by Chaudron et al. (2004) and Carroll et al. (2013), care was delivered by the pediatric staff from a primary care center. In the Norwegian Glavin et al. study (2010, 2012), public health nurses screened the mothers at well-baby clinics, a comparable setting to that of the Leung et al. (2011) study in Hong Kong, where nurses screened the mothers at Maternal and Child Health Centers. The screening investigated by Gerrard et al. (1993) was carried out by trained health visitors at baby clinics in England. Yawn et al. (2012) focused on family medicine research network practices in 21 USA states; 22 of the included practices offered continuity to the mother and her child, and six only to the mother. Pediatrician offices offering services only to the child were excluded. Except for the six practices studied by Yawn et al., the other practices offered frequent appointments to both mother and child. In the first year postpartum the frequency varied from 7 to 10. The intention of the settings was to service the general population and to reach 90–100 % of the mothers of newborn children in their area. The frequency and outreach of the services in the Gerrard et al. study (1993) could not be verified.

### Intervention Content

The interventions offered in the various studies differed greatly. Those in the Chaudron et al. (2004) and Carroll et al. (2013) studies consisted mainly of incorporating screening questionnaires into the regular visits. In addition, Carroll et al. used a decision support system, incorporated in an electronic medical support system. Depending on the answers on the screening questionnaire, reminders were created by the system to guide clinicians during their visit. Four of the six studies (Yawn et al. 2012; Glavin et al. 2010; Leung et al. 2011; Gerrard et al. 1993) investigated an intervention consisting of both screening and enhanced care. In the Glavin et al. study (2010) screening was one of several components of the intervention and was followed by a standard supportive counseling session for all mothers with the Public Health Nurse. Depressed mothers received follow-up supportive counseling sessions. Yawn et al. (2012) compared a practice-based training program for screening, diagnosis, and management of mothers with PPD. Intervention practices were provided with a set of tools to facilitate each part of the process. Leung et al. (2011) also described the steps following screening: participants with a positive EPDS were directed to another nurse for counseling. During this session, subsequent management was recommended. This could be either non-directive counseling by a Maternal and Child Health Centre (MCHC) nurse or referral to the community psychiatric team. These steps were also offered to mothers clinically observed as depressed, and were therefore not limited to the intervention. Mothers with elevated EPDS scores in the post-training group of the Gerrard et al. study (1993) were offered 4–8 non-directive counselling visits by their health visitor.

### Screening Instrument, Cut-off Score and Timing

Five studies used the EPDS as the screening instrument; four (Yawn et al. 2012; Glavin et al. 2010; Chaudron et al. 2004; Leung et al. 2011) had the same cut-off score of ≥10 and one, by Gerrard et al. (1993), selected 12 as the cut-off score. Glavin et al. (2010) and Chaudron et al. (2004) mentioned that clinical judgment should confirm the EPDS indication of a mother as probably being depressed. Leung et al. (2011) also considered a positive answer on question ten (suicidal ideation) as indicative. Carroll et al. (2013) adapted a validated two question depression screening tool into an existing pre-screening form. In the study by Yawn et al. (2012), mothers with an EPDS score of ≥10 were asked to complete the Patient Health Questionnaire (PHQ-9) as well. A mother was considered to have PPD if her PHQ-9 score was ≥10 and the physician’s evaluation revealed no other cause for the depressive symptoms. Carroll et al. (2013) reported the PHQ-9 was added as a hand-out to one of the two intervention arms to assist the physician in diagnosing depression but no PHQ-9 data were shown in the results. In the studies by Leung et al. (2011), Glavin et al. (2010) and Yawn et al. (2012), screening was performed once, at 2 months, 6 weeks and between 5–12 weeks postpartum, respectively. In the Chaudron et al. study (2004), mothers received the EPDS at each well-child visit during the child’s first year, starting with the routine 2 week visit. In the study by Carroll et al. (2013), mothers were screened every 3 months until the age of 15 months. Health visitors in the Gerrard et al. study (1993) were instructed to screen at 6–8 weeks and/or 10–12 weeks, depending on the number of training sessions attended by the health visitor.

### Outcome Measures

The types of primary outcomes depended on the study design. Studies examining screening without enhanced care (Chaudron et al. 2004; Carroll et al. 2013) used documented depressive symptoms and referrals, indicated in Table 2 as primary outcomes at process level. Five studies (Yawn et al. 2012; Glavin et al. 2010; Chaudron et al. 2004; Leung et al. 2011; Carroll et al. 2013) reported the rates of the elevated scores on their screening instrument at the moment of intervention. None of the studies used a golden standard to confirm the PPD diagnosis. The four studies (Yawn et al. 2012; Glavin et al. 2010; Leung et al. 2011; Gerrard et al. 1993), which examined screening combined with enhanced care, used the screening instrument of their intervention also as a primary outcome measure for maternal depressive symptoms later in the postpartum year. Regarding secondary outcomes, different outcome measures were used. Three (Yawn et al. 2012; Glavin et al. 2010; Leung et al. 2011) of those studies used the Parenting Stress Index (PSI). The only secondary outcome at child level was the child’s body weight at 6 and 18 months presented by Leung et al. (2011).

### Study Quality

Table 3shows the outcomes of the Quality Assessment tool for Quantitative Studies (Armijo-Olivo et al. 2012).

**Table 3:** Quality of the 6 included studies, assessed with the Quality Assessment tool for Quantitative Studies (Armijo-Olivo et al. 2012)

	Gerrard et al. (1993)	Chaudron et al. (2004)	Glavin et al. (2010)	Leung et al. (2011)	Yawn et al. (2012)	Carroll et al. (2013)
Selection bias	Weak	Strong	Strong	Moderate	Strong	Strong
Study design	Moderate	Weak	Moderate	Strong	Strong	Strong
Confounders	Weak	Weak	Weak	Strong	Strong	Weak
Blinding	Weak	Moderate	Moderate	Moderate	Moderate	Strong
Data collection method	Strong	Weak	Strong	Strong	Strong	Weak
Withdrawals and dropouts	Weak	Not applicable	Weak	Moderate	Moderate	Not applicable
Global rating	Weak	Weak	Weak	Strong	Strong	Weak

Four (Glavin et al. 2010; Chaudron et al. 2004; Carroll et al. 2013; Gerrard et al. 1993) of the six studies were globally rated as weak, according to this Quality Assessment tool. All four studies had a weak score on description and control of possible confounders. In both Chaudron’s (Chaudron et al. 2004) and Carroll’s (Carroll et al. 2013) study the data collection methods were weak as their data were based on health care provider documentations, which were incomplete and not based on valid instruments in the control groups.

### Interpretation of Results

Four studies presented screening outcomes at process level (Table 2) (Yawn et al. 2012; Chaudron et al. 2004; Leung et al. 2011; Carroll et al. 2013). The effect on the detection rate when screening for PPD was quantified in three of the six studies (Chaudron et al. 2004; Leung et al. 2011; Carroll et al. 2013). The calculated RRs for detection of PPD in the studies by Chaudron et al. (2004) and Leung et al. (2011) were, respectively, 5.3 (8.5 %/1.6 %) and 4.8 (29 %/6 %). Improvement in the rate of referral in the study by Carroll et al. (2013) was presented with an OR of 2.06 (95 % confidence interval (CI) 1.08–3.93). We calculated the RRs for the other three studies: for the referral to a social worker in the study by Chaudron et al. (2004) the RR was 18 (3.6/0.2), for receiving treatment in the study by Leung et al. (2011) the RR was 4.9 (23.8/4.8), and for being diagnosed as PPD in the study by Yawn et al. (2012) the RR was 1.6 (66 %/41 %). Carroll et al. (2013) mentioned that adding handouts to the screening process resulted in earlier referral, but no data were presented.

Four of the six studies (Yawn et al. 2012; Glavin et al. 2010; Leung et al. 2011; Gerrard et al. 1993) (including the two strong studies) in which screening and enhanced care were combined in the intervention, showed significant improvement of depression scores later in the postpartum year in the intervention arms. In the Leung et al. study (2011), mothers in the intervention group had an RR of 0.59 (95 % CI 0.39–0.89) for having an elevated EPDS (≥10) at 6 months postpartum. In the Glavin et al. study (2010), mothers in the intervention group had an OR of 0.5 (95 % CI 0.3–0.8) for having an elevated EPDS (≥10) and in the Gerrard et al. study (1993) the post-training group had an RR of 0.51 (9.8 %/19.3 %) for an EPDS of 12 or above. Mothers in the intervention group in the Yawn et al. (2012) study had an OR of 1.74 (95 % CI 1.05–2.86) for having a ≥5-point drop in PHQ-9 score between baseline and 12 months postpartum. Of the mothers in the study of Glavin et al. (2010) who had an EPDS score of 10 or above at 6 weeks postpartum, those in the intervention group had a larger improvement in EPDS scores from 6 weeks to 12 months postpartum compared to the those in the control group (effect size 0.53). We could not create a summarized effect size as the measurement moments and outcome measures in the included six studies varied too much.

Regarding secondary outcomes, there were no results on child development or social-emotional wellbeing. No significant difference was found with respect to the child’s weight in the Leung et al. study (2011). At parent level, no statistical significant differences were found in secondary outcomes regarding measuring long-term effects (Table 2), except in the study by Glavin et al. (2010). The intervention group’s PSI Health subscale 12 months postpartum demonstrated a better score.

## Discussion

This review has identified limited but promising evidence for the effectiveness of screening for PPD on maternal health outcomes. Four (Yawn et al. 2012; Chaudron et al. 2004; Leung et al. 2011; Carroll et al. 2013) of the six studies indicate an increase in detection rate of depressive symptoms or referral or treatment rates and four studies report a reduction in depressive symptoms at 3, 6 or 12 months postpartum (Glavin et al. 2010; Leung et al. 2011; Gerrard et al. 1993; Yawn et al. 2012). Screening on PPD leads to significant changes in the measured secondary outcomes at mother level in only one study; no relevant outcomes were measured at child level. Both strong quality studies were conducted in a setting providing care for both mother and child, with an intervention consisting of a combination of screening with some enhancement of care. It was not possible to untangle the effect of screening from the offer of extra care.

The improvement in depression scores, and yet the lack of the effect on secondary outcomes is comparable with studies on screening for PPD in general. In the HTA-review of Hewitt et al. (2009) outcomes were combined. This resulted in a pooled OR of 0.64 (95 % CI 0.52–0.78) for scoring above the threshold for depression for women in an intervention group compared to the control group. This effect is comparable to those demonstrated by Leung et al. (2011) and Yawn et al. (2012). The HTA review also encountered the same problem of disentanglement regarding the effect of screening and enhancement of care, and the lack of evidence of improving other maternal and child outcomes. The Agency for Healthcare Research and Quality (AHRQ) report (Myers et al. 2013) selected some of the same studies as our review, and also concludes that screening has a positive effect on depressive symptoms, but effects on secondary outcomes have not been proven.

The included studies may not have fully exploited the potentials of screening for PPD in WBC, for several reasons. One aspect is the timing of screening; the potential benefit of screening in a WBC setting may lie mainly in the possibility of repeated screening and continuous follow-ups. However, only three (Chaudron et al. 2004; Carroll et al. 2013; Gerrard et al. 1993) (weak quality) studies had repeated screening interventions. Furthermore, mothers in the control group of other studies (Yawn et al. 2012; Leung et al. 2011), with high scores on the screening instrument or suicidal thoughts at the time of intervention, were also given follow-up advice for ethical reasons. This may have reduced the effect of the intervention on secondary outcomes.

Another factor influencing the secondary outcomes may have been the follow-up-process after screening. Recent studies (Myers et al. 2013; Yawn et al. 2012) advise to incorporate follow-up care within the same (primary care) setting as the screening, which is the case in the two strong studies (Yawn et al. 2012; Leung et al. 2011). Although significantly more mothers in the intervention groups were diagnosed and/or treated, a substantial number of the depressed women did not receive this follow-up care. As a consequence, screening might have been less effective. Finally, most of the included studies used ≥10 as the EPDS screening cut-off score. According to Hewitt et al. (2009) this is the optimal cut point if screening for both major and minor depression, while 12 is optimal if screening for major depression only. Use of different cut points may affect the effectiveness of screening.

Only one study measured the effect of screening for PPD at child level by including the child’s weight. As the effect of PPD on the child’s wellbeing is an important argument in favor of the necessity of screening, we expected studies examining both screening and enhanced care to also include some outcomes at child level. Possible explanations for not including outcomes at child level might be the limited options for standardization of the quality of care after screening and for measuring social-emotional development in the first year after birth. In addition, controlling the moderators and mediators influencing the social-emotional development is difficult.

### Strengths and Limitations

Although many countries have preventive child health care incorporated in their health care system, nomenclature proved to be quite diverse. We carefully identified the different options to ensure we included the most relevant articles in our search. Another strength of our review is the thorough systematic search of three extensive databases, supplemented by systematic hand searches of reviews included in the search. Every step of the selection process was consistently executed and judged by two independent reviewers.

A limitation may be that we did not search the grey literature for evidence, thus some relevant studies may have been missed. Reporting bias may have influenced the outcomes of this review, as the studies included in the review only reported the positive effect of screening.

### Implications

Screening for postpartum depression calls for a setting that has the facility to combine screening with the judgment of a professional, reaches most new mothers, has professionals available who are in a position to create a bond of trust, and offers frequent contact to the mother in the first year postpartum. Professional preventive services for child healthcare can meet all of these criteria, and our current review supports the potential of screening in WBC with positive evidence. The small number of studies limits the precision of the effect estimates.

Future research should aim at creating stronger evidence of the possible benefits of this combination of characteristics when screening in a WBC setting. General aspects of the design and intervention need attention, such as cut-off scores, golden standards to be used, a control group and the possibility of separating the effect of screening and subsequent offers of extra care. Moreover, new research should explore the benefits of repeated screening during the first year postpartum and, preferably, also include outcomes at child level.

## Conclusions

The evidence in this review on the effectiveness of screening for PPD in a WBC setting is promising, though based on a limited number of studies. The use of a validated instrument like the EPDS led, in all the included studies, to significantly higher detection of mothers with depressive symptoms or, when screening was combined with enhanced care, to improvement of depression scores. Whether this leads to better outcomes for mother and child on the long term needs additional high-quality research. The potential health gains of screening for PPD in a WBC setting are large but need to be confirmed.

## Appendix 1: Final Search Terms in SCOPUS, Listed Per Topic

### Postpartum Depression

((TITLE-ABS-KEY(postpartum OR postnatal OR perinatal OR “after birth” OR puerperal) AND TITLE-ABS-KEY(depress* OR mood)))

### Screening

AND (TITLE-ABS-KEY(screening OR screen OR screened OR identif* OR “at risk” OR preventi* OR interven* OR recogni* OR “depression scale” OR tool OR program* OR strategy))

### Well-Baby Care Setting

AND (TITLE-ABS-KEY(pediatr*) OR TITLE-ABS-KEY(paediatr*) OR TITLE-ABS-KEY(well child) OR TITLE-ABS-KEY(“well-child”) OR TITLE-ABS-KEY(“well baby”) OR TITLE-ABS-KEY(“well-baby”) OR TITLE-ABS-KEY(“youth health care”) OR TITLE-ABS-KEY(“child health care”) OR TITLE-ABS-KEY(“home visit*”) OR TITLE-ABS-KEY(“health visit*”) OR TITLE-ABS-KEY(“maternal and child health”) OR TITLE-ABS-KEY(“maternal child health”) OR TITLE-ABS-KEY(“primary care”) OR TITLE-ABS-KEY(“primary health care”) OR TITLE-ABS-KEY(“public health”) OR TITLE-ABS-KEY(“community health”) OR TITLE-ABS-KEY(“postpartum care”) OR TITLE-ABS-KEY(“maternal care”) OR TITLE-ABS-KEY(“perinatal care”) OR TITLE-ABS-KEY(“perinatal health services”))

### Excluded Subject Areas

AND (EXCLUDE(SUBJAREA, “NEUR”) OR EXCLUDE(SUBJAREA, “BIOC”) OR EXCLUDE(SUBJAREA, “NEUR”) OR EXCLUDE(SUBJAREA, “BIOC”) OR EXCLUDE(SUBJAREA, “PHAR”) OR EXCLUDE(SUBJAREA, “AGRI”) OR EXCLUDE(SUBJAREA, “ENVI”) OR EXCLUDE(SUBJAREA, “IMMU”) OR EXCLUDE(SUBJAREA, “BUSI”) OR EXCLUDE(SUBJAREA, “CENG”) OR EXCLUDE(SUBJAREA, “DENT”))

## Abbreviations

PPD: Postpartum depression
WBC: Well baby care
EPDS: Edinburgh Postnatal Depression Scale
RCT: Randomized controlled trial
GP: General practitioner
MCHC: Maternal and Child Health Center
MCH: Maternal and child health
PHQ-9: Patient Health Questionnaire
PSF: Pre-screening form
JIT: ‘Just in time’ handout
PSI: Parenting Stress Index
GHQ-12: 12-Item General Health Questionnaire
CKMSS: Chinese Kansas Marital Satisfaction Scale
DAS-6: Dyad Adult Satisfaction short form
OR: Odds ratio
CI: Confidence interval
RR: Risk ratio
